# Efficient Detection of Long dsRNA *in Vitro* and *in Vivo* Using the dsRNA Binding Domain from FHV B2 Protein

**DOI:** 10.3389/fpls.2018.00070

**Published:** 2018-02-01

**Authors:** Baptiste Monsion, Marco Incarbone, Kamal Hleibieh, Vianney Poignavent, Ahmed Ghannam, Patrice Dunoyer, Laurent Daeffler, Jens Tilsner, Christophe Ritzenthaler

**Affiliations:** ^1^Centre National de la Recherche Scientifique, Institut de Biologie Moléculaire des Plantes, Université de Strasbourg, Strasbourg, France; ^2^Centre National de la Recherche Scientifique, Institut de Biologie Moléculaire et Cellulaire, Université de Strasbourg, Strasbourg, France; ^3^Biomedical Sciences Research Complex, University of St Andrews, St Andrews, United Kingdom; ^4^Cell and Molecular Sciences, The James Hutton Institute, Dundee, United Kingdom

**Keywords:** dsRNA, virus, viral factories, replication complexes, detection, plant, insect, *Nicotiana benthamiana*

## Abstract

Double-stranded RNA (dsRNA) plays essential functions in many biological processes, including the activation of innate immune responses and RNA interference. dsRNA also represents the genetic entity of some viruses and is a hallmark of infections by positive-sense single-stranded RNA viruses. Methods for detecting dsRNA rely essentially on immunological approaches and their use is often limited to *in vitro* applications, although recent developments have allowed the visualization of dsRNA *in vivo*. Here, we report the sensitive and rapid detection of long dsRNA both *in vitro* and *in vivo* using the dsRNA binding domain of the B2 protein from Flock house virus. *In vitro*, we adapted the system for the detection of dsRNA either enzymatically by northwestern blotting or by direct fluorescence labeling on fixed samples. *In vivo*, we produced stable transgenic *Nicotiana benthamiana* lines allowing the visualization of dsRNA by fluorescence microscopy. Using these techniques, we were able to discriminate healthy and positive-sense single-stranded RNA virus-infected material in plants and insect cells. In *N. benthamiana*, our system proved to be very potent for the spatio-temporal visualization of replicative RNA intermediates of a broad range of positive-sense RNA viruses, including high- vs. low-copy number viruses.

## Introduction

Double-stranded (ds)RNA that results from the pairing in *cis* or in *trans* of two complementary RNA strands has been postulated to be the earliest form of life (Gilbert, 1986; Joyce, 1989). Long dsRNA is also regarded as a universal hallmark of infection of cellular organisms by viruses (Morris and Dodds, 1979; Kawai and Akira, 2006). In this respect, double-stranded RNA viruses have dsRNA as their genome. Single-stranded RNA viruses produce dsRNA in the form of intermediates of replication as a direct result of the viral RNA-dependent RNA polymerase (RdRP) activity and indirectly due to host-encoded RNA-dependent RNA polymerases. With DNA viruses, dsRNA production generally results from the pairing of converging overlapping transcripts of the viral genome. Finally, dsRNA can also be generated by secondary structures in viral transcripts.

Viruses being pathogenic in essence and considering their intrinsic capacity to produce dsRNA during their replication cycle, a set of sophisticated innate immune defense mechanisms that specifically involve dsRNA recognition have emerged during evolution, such as RNA silencing (Takeuchi and Akira, 2009; Goubau et al., 2013). RNA silencing is a well conserved biological process found in most eukaryotic cells that inhibits gene expression at the transcriptional (transcriptional gene silencing; TGS) or post-transcriptional (post-transcriptional gene silencing PTGS) level through sequence-specific interactions. This phenomenon is of prime importance in antiviral defense but has other biological functions in genome stability and development (Plasterk, 2002; Ghildiyal and Zamore, 2009; Holoch and Moazed, 2015). The initial step of RNA silencing depends on the recognition of a long dsRNA by an RNase III-like enzyme called Dicer (or DCL, for Dicer-like, in plants) that processes the trigger dsRNA into small RNA duplexes of 21–24 nt in length, with 2 nt 3′ overhangs. In vertebrates, insects and worms, long stretches of dsRNA are not readily found under healthy conditions and are considered as danger signals or microbe-associated molecular patterns (MAMPs) that trigger other types of innate immune reactions (de Faria et al., 2013; Ermolaeva and Schumacher, 2014; Mussabekova et al., 2017). There, long dsRNA is recognized by intracellular or extracellular pathogen recognition receptors such as Toll-like receptor (TLR), retinoic acid-inducible gene 1 (RIG-I) and melanoma differentiation-associated protein (MDA5) that activate signaling pathways resulting in the biosynthesis of antiviral compounds including antimicrobial peptides, type I interferon and/or inflammatory cytokines (Akira et al., 2006; Schneider et al., 2014; Mussabekova et al., 2017).

In view of the central role played by dsRNA in many fundamental biological processes, a whole range of approaches have been developed to detect or visualize dsRNA *in vitro* or *in situ* (Morris and Dodds, 1979; Poynter and DeWitte-Orr, 2017). The majority of these, such as immunofluorescence, ELISA, immunoblot, etc., rely on immunological methods where antibodies are used as structural probes that specifically recognize the A-helix structure adopted by dsRNA (Moffitt and Lister, 1975; Aramburu et al., 1991; Powell, 1991; Schönborn et al., 1991; Lee et al., 1994; O'Brien et al., 2015; Son et al., 2015). Over time, the commercially available J2 anti-dsRNA IgG2a and to a lesser extent the IgG2a K1 and IgM K2 mAb (Schönborn et al., 1991) have become the golden standards in dsRNA detection and have been used extensively in a wide range of systems, as documented in over 400 scientific publications (scicons.eu/en/antibodies/j2/). However, in a recent study, Son et al. (2015) showed the superiority of the new monoclonal antibody (mAb) 9D5 over mAb J2 for the detection of dsRNA from negative-stranded and ambisense RNA viruses, as well as single-stranded DNA viruses. Besides immunological detection, antibody-independent methods such as nucleic acid fluorescent *in situ* hybridization (FISH) (Bolten et al., 1998; Montgomery et al., 1998) or cellulose-based dsRNA isolation (Morris and Dodds, 1979) have also been used for dsRNA detection, but all these are incompatible with the spatio-temporal visualization of dsRNA *in vivo*. To circumvent this major limitation, Cheng et al. (2015) recently made use of the specific dsRNA binding property of three viral dsRNA binding proteins (DRBs), NS1, B2, and VP35 from influenza A virus (Bornholdt and Prasad, 2008), Flock house virus (FHV) (Chao et al., 2005; Lingel et al., 2005) and Marburg virus (MARV)(Bale et al., 2012), respectively, to develop a dsRNA binding-dependent fluorescence complementation assay (dRBFC). Upon transient co-expression of the DRBs fused to N- and C-terminal fragments of the yellow fluorescent protein (YFP), they managed to visualize host structures involved in RNA silencing as well as replicative RNA intermediates of positive-sense RNA viruses in living *Nicotiana benthamiana* leaf protoplasts or directly in agro-infiltrated leaf tissues (Cheng et al., 2015).

Here, we report about *in vitro* and *in vivo* methods for the sensitive and specific detection of long viral dsRNA based on the use of the dsRNA binding domain from FHV B2 protein (Chao et al., 2005). *In vitro*, we adapted the system for the detection of dsRNA by northwestern blotting or by direct fluorescence labeling of fixed samples. *In vivo*, we produced stable transgenic *N. benthamiana* lines allowing the direct visualization of dsRNA from various RNA viruses in a simple, reproducible and dynamic manner.

## Materials and methods

### Reagents

The following reagents were purchased: mouse mAb J2 (Scicons); Alexa Fluor 488-goat anti-mouse IgG secondary antibody (Molecular Probes, Life Technologies); Phi6 dsRNA (Finnzymes, Thermo Fisher Scientific); dsRNA ladder (New England Biolabs); High MW poly (A:U) and poly (I:C) (InvivoGen); GeneRuler 1 kb DNA Ladder (Thermo Fisher Scientific); *Silencer* negative control 1 siRNA (Ambion, Life Technologies); Strep-Tactin conjugated to horseradish peroxidase (IBA Lifesciences); pNPP (Sigma-Aldrich), mMESSAGE mMACHINE T7 transcription kit (Ambion, Life Technologies), Lumi-Light western blotting substrate (Roche Diagnostics).

### Virus strains

Grapevine fanleaf virus (GFLV isolate GHu), Turnip crinkle virus (TCV) and Tobacco rattle virus (TRV) are described in Vigne et al. (2013), Thomas et al. (2003), and Liu et al. (2002), respectively. Tomato bushy stunt virus (TBSV) was generously provided by Bioreba AG, Switzerland. PVX.TagBFP-CP was a generous gift of C. Dickmeis and U. Commandeur (RWTH Aachen University, Germany). PVX.GFP-CP, PVX.mCherry-CP and PVX.ΔTGB1.GFP-CP have been previously described (Santa Cruz et al., 1996; Tilsner et al., 2009, 2012, 2013; Linnik et al., 2013). All PVX constructs include a Foot and mouth disease virus 2A peptide linker between the fluorescent protein and the CP, which results in the translation of a mixture of fused and free CP to enable virus encapsidation (Santa Cruz et al., 1996). All PVX constructs are under control of a Cauliflower mosaic virus 35S promoter, and were inoculated by microprojectile bombardment as previously described (Linnik et al., 2013). Fluorescent fusions of viral proteins and the Pumilio-BiFC ssRNA imaging system have been previously described (Tilsner et al., 2009, 2012, 2013) and were introduced by agroinfiltration as described in Tilsner et al. (2012). Turnip mosaic virus (TuMV)-mCherry binary plasmid was a generous gift of J. F. Laliberté (INRS-Institut Armand-Frappier, Laval, Canada). Drosophila C virus (DCV) was produced in and purified from drosophila S2 cells (Jousset et al., 1977; Johnson and Christian, 1998).

### Cloning procedures

The nucleotide sequence of Flock house virus (GenBank accession X77156) coding for B2 amino acids 1 to 73 was amplified by PCR using primer pair #1 and #2 in order to add a His_6_ tag and a Trp-Ser-His-Pro-Gln-Phe-Glu-Lys StrepTag II sequence (Schmidt and Skerra, 2007). The amplicon was inserted into NdeI and XhoI digested pET-22b(+) plasmid (Merck Millipore) to obtain pET22-B2HS plasmid encoding protein named B2 hereafter. Former full-length pET22-B2HS plasmid was amplified with Phusion High-Fidelity DNA Polymerase (ThermoScientific) using primer pair #3 and #4, then template was digested by DpnI and amplicon was ligated to generate plasmid pET22-B2mHS encoding a mutated B2 with 2 substitutions (C44S and K47A) named B2m hereafter.

In-frame fusions of B2 (aa 1-73) with EGFP- or TagRFP-His_6_ tag ± StrepTag II separated by a GlyGlyGlySerGlyGlyGly linker named respectively B2:GFP and B2:RFP hereafter, were obtained by overlap extension PCRs using for B2:GFP the primer pairs (#5 and #6) in combination with primer pairs (#7 and #8) and for B2:RFP the primer pairs (#5 and #9) in combination with primer pairs (#10 and #11). The final common overlapping PCR was performed using primer pair (#5 and #12).

The resulting PCR products were inserted into pDONR/Zeo vector using Gateway BP Clonase II Enzyme mix (Invitrogen), then introduced into a pEAQ HT Dest1-derived vector (Sainsbury et al., 2009) named pEAQΔP19 in which P19 was deleted, or into p0GWAΔ_RBS_ using Gateway LR Clonase Enzyme mix (Invitrogen) following the supplier's instructions. p0GWAΔ_RBS_ destination vector was obtained from original p0GWA (Busso et al., 2005) by deletion of the XbaI-KpnI fragment containing ribosome binding site (RBS) and start codon upstream AttR1 recombination site. Subsequently, PCR products containing a RBS 7 base pairs upstream B2 gene initiation codon were obtained using primer pairs (#13 and #12) for His_6_ tag and StrepTag II fusion or primer pairs (#13 and #14) for His_6_ tag fusion only and were introduced into Donor vector in order to express proteins without a AttB1 N-terminal fusion. The coding nucleotide sequences of the entire set of clones were verified. Sequences of primers used for PCR are provided in Table S1.

### *Nicotiana benthamiana* transformation

*Nicotiana benthamiana* transformation using Agrobacterium *tumefaciens* GV3101::pMP90 was performed as described in Hemmer et al. (2017). Regenerated T0 plants were self pollinated through T2 generation. As many lines supposedly harbor multi-insertions, wild-type *N. benthamiana* flowering plants were crossed with transgenic pollen from B2:GFP and B2:RFP T2 lines in order to obtain single insertion transgenic lines. Single insertion status was determined by fluorescence-based segregation among generations. Homozygous plants were not fertile and selection of expressing plants performed on seedling by epifluorescence microscopy.

### Protein expression and purification

Expression was performed in freshly transformed *E. coli* BL21 (DE3) cells grown in Terrific Broth medium and induced overnight with 0.5 mM IPTG at 20°C. Pelleted cells resuspended in phosphate buffer saline (PBS)-NaCl (PBS 1X, 1 M NaCl, pH 7.4) were lysed by sonication [80% amplitude for 150 s with 13 mm diameter probe, Vibra-Cell VCX 500 (Sonics)]. Tagged proteins were purified at 4°C by immobilized metal ion chromatography (IMAC) on a 1 ml Protino Ni-NTA column (Macherey-Nagel) using 500 mM imidazole in running buffer (50 mM Tris, 300 mM NaCl, 5% glycerol, pH 8.0) for elution, followed by size exclusion chromatography (SEC) on a Hiload 16/60 Superdex75 prep grade column (GE Healthcare Life Science) in 1X PBS. Purity of eluted proteins was assessed by Coomassie blue staining of protein separated on denaturing Tris-tricine or Tris-glycine polyacrylamide gels. Purification yields were estimated from absorbance at 280 nm based on extinction coefficients computed from protein amino acid composition. Purified proteins were adjusted to 1 mg.ml^−1^ in 50% glycerol and stocked at −20°C.

### Nucleic acids

Single-stranded RNA (ssRNA) was obtained by *in vitro* transcription of the “gf” fragment from the Green Fluorescent Protein as described in Montavon et al. (2017); dsRNA production by *in vitro* hybridization of the “gf” and “fg” fragments from GFP is also described in Montavon et al. (2017). B2 PCR amplicon using pET22-B2HS plasmid as template was used as dsDNA control and denatured amplicon as ssDNA control.

### Electrophoretic mobility shift assay (EMSA)

Nucleic acid binding activity was evaluated by EMSA. Binding reactions were performed by incubating 300 ng of nucleic acids with 100 pmoles of purified B2 proteins in 0.5X TBE buffer containing 100 mM NaCl at room temperature for 15 min. After incubation, the products of the binding reaction were resolved by native 1% agarose gel electrophoresis at 10 V.cm^−1^ at 4°C, except for EMSA with dsRNA ladder (1.5 μg dsRNA + 300 pmoles of B2 purified protein per lane) resolved through a 12% polyacrylamide 19:1 gel in 1X TBE buffer containing 100 mM NaCl and 5% glycerol. Nucleic acids were visualized after ethidium bromide staining, using E-BOX VX5 gel imaging system (Vilber Lourmat), and fluorescent B2 protein was detected using G:BOX Chemi XRQ fluorescence imaging system (Syngene).

### Protoplast preparation and immunofluorescence staining

Protoplasts were isolated and transfected as described in Wu et al. (2009). Two days post-transfection, protoplasts were washed and resuspended in M&Ms buffer [0.55 M Mannitol, 5 mM MgCl_2_ and 2 mM 2-(*N*-morpholino)ethanesulfonic acid (MES)], then fixed in 2% paraformaldehyde (PFA) and 1% glutaraldehyde final concentrations in 4 mM MES and 10 mM EGTA for 20 min before adding 0.05% Triton-X100 for 10 min. Protoplasts were washed once in M&Ms buffer and three times in increasing PBS concentrations from one third to 1X. Protoplasts were immobilized on poly-L-lysine pre-coated coverslips and treated overnight with 0.1% sodium borohydride in 1X PBS as described in Ritzenthaler et al. (2002b). Unspecific binding was blocked by incubation in PBST (PBS 1X, 0.05% Tween 20) containing 5% skimmed milk powder for 4 h at 4°C. Primary antibodies for detection of dsRNA (J2, 1/200 dilution) and purified B2:RFP (6.7 μg.ml^−1^) were incubated overnight at 4°C, washed for 5 min in PBS three times before incubation with Alexa Fluor 488 goat anti-mouse IgG (1/300 dilution, used for the detection of the J2 mAb) for 2h, and finally washed with PBST for 5 min three times, twice with PBS for 5 min and once with distilled H_2_O for 1 min. Microscope slides were mounted and viewed by confocal microscopy.

### Coexpression of B2:GFP with of the mitochondrial marker F_0_-ATPase:RFP

To assess co-localization of TRV replication complexes with mitochondria, leaves of B2:GFP *N. benthamiana* reporter plants were infiltrated with *A. tumefaciens* carrying pSu9 encoding the mitochondrial marker F_0_-ATPase:RFP (Sieber et al., 2011; Michaud et al., 2014) through a pH7RGW2 binary plasmid, at OD_600nm_ 0.2. Shortly after inoculation, the same leaves were infected through rub inoculation with a crude extract from TRV-infected *A. thaliana* in 50 mM sodium phosphate buffer pH 7.5. Observation of the infected leaves was carried out 4 days post-inoculation (dpi).

### DCV infection

Drosophila S2 cells were grown on slides (Millicell EZ, Merck, Germany) in Schneider medium complemented with 10% fetal calf serum (FCS), 1% glutamax and 1% Penicillin/Streptomycin. Cells were infected with DCV at a multiplicity of infection (moi) of 1 PFU/cell, or mock-treated, using serum free Schneider medium for 1 h. The inoculum was then removed and fresh complete Schneider medium was added for further 24 h onto the cultures.

For immunofluorescence, S2 cells were fixed for 30 min using a 1x PBS solution containing 4% PFA. Next, cells were permeabilized twice, for 5 min at RT, using PBT (1X PBS, 0.1% Triton-X100) solution. Blocking of non-specific sites was performed by incubating the permeabilized cells for 30 min under gentle shaking in PBT supplemented with 10% FCS. The solution was then discarded and the mouse mAb against dsRNA, J2, was applied (1:200 dilution) in PBT solution supplemented with 10% FCS onto the cells overnight at 4°C under gentle shaking. The next day, cells were washed twice with PBT and further incubated at RT for 2 h with a PBT solution containing an Alexa Fluor 488 donkey anti-mouse antibody (1:600 dilution) and purified B2:RFP (20 μg.ml^−1^). Cells were then washed twice in PBT, covered with Vectashield mounting medium containing 4′,6-diamidino-2-phenylindole (DAPI; Vector Laboratories) and examined by confocal microscopy.

For northwestern blotting, RNA was extracted from cells using Tri Reagent (Molecular Research Central) according to the manufacturer's instructions.

### Confocal laser scanning microscopy

Imaging was performed on a Zeiss LSM710 Meta, Zeiss LSM700 or a Leica SP2 confocal laser scanning microscope. BFP was excited at 405 nm, EGFP and GFP at 488 nm, mCitrine (Pumilio-BiFC) at 514 nm, TagRFP at 561 nm, and mCherry at 594 or 561 nm. Detection ranges were optimized for each fluorophore combination, and all images were obtained in sequential scanning mode to minimize bleed-through. Microscope power settings were adjusted to optimize contrast for each individual image. Figures were assembled in Adobe Photoshop CS5. For long-term observations of PVX infection, dsRNA sensor transgenic *N. benthamiana* plants were imaged at 4 days post inoculation by gluing inoculated leaves onto microscope slides with double-sided sticky tape, keeping the leaf attached to the plant, which was held beside the microscope stage on a height-adjustable stand. To further minimize leaf movements on the slide, two bands of sticky tape were wrapped around both leaf and slide at either side of the imaged lesion. A 20x long-distance lens was used to avoid using water as an immersion fluid which would have had to be replenished at regular intervals. The time series and tile scanning functions of Zeiss ZEN software package were used to program automatic acquisition of a 3-by-3 tile (corresponding to 1.2 × 1.2 mm leaf area) z-stack every 1 h. Z-stack upper and lower boundaries were set approximately 10 μm above and below the epidermal cell layer to account for the uneven leaf surface and the possibility of vertical tissue movement due to uneven adhesion to the sticky tape. Acquisition of each tile scan stack took approximately 40 min, thus the z-series at every tile position was completed before moving to the next tile.

### Northwestern blotting

Total RNA samples were electrophoresed at 4°C in 12% polyacrylamide 19:1 gels for siRNA and dsRNA ladder or in 1% agarose gels containing either 0.5X TBE buffer (44.5 mM Tris HCl, 44.5 mM boric acid, 1 mM EDTA, pH 8) or 1X HEPES buffer (20 mM HEPES, 1 mM EDTA, pH 7.4). Capillary transfer of nucleic acids was performed overnight onto a Hybond-N^+^ nylon membrane (GE Healthcare) with SSC 20X (3 M NaCl, 300 mM trisodium citrate adjusted to pH 7.0 with HCl) before UV-crosslink (Crosslinker UVC5000, Hoefer). Membranes were incubated in 5% skimmed milk powder for 2 h at room temperature to block nonspecific binding sites, incubated with a 3.5 μg.ml^−1^ B2 final concentration in PBST-milk for 1 h at room temperature, washed in PBST for 10 min three times and then incubated with a 1:5,000 dilution of Strep-Tactin conjugated to horseradish peroxidase from (IBA Lifesciences) in PBST-milk for 1h at room temperature. After washing in PBST for 10 min three times, membranes were incubated with Lumi-Light western blotting substrate (Roche Diagnostics) and revealed with Fusion FX imaging system (Vilber Lourmat) or Fujifilm autoradiographic films.

## Results

### B2 and B2:FP specifically recognize dsRNA *in vitro*

**Figure 1:**
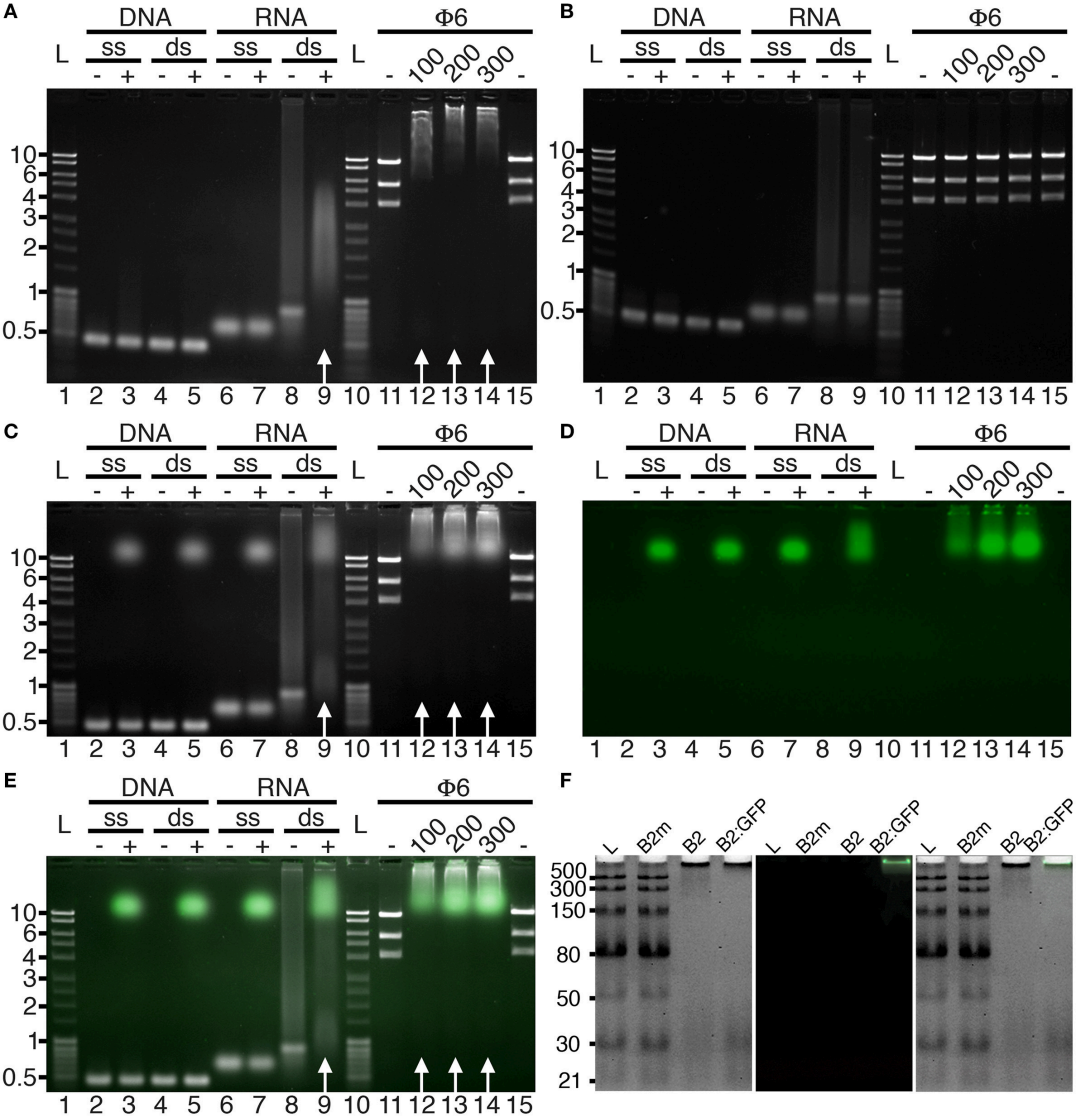
Binding specificity of B2 and B2:GFP *in vitro*. B2 **(A,F)**, B2m **(B,F)**, and B2:GFP **(C–F)** were tested by EMSA for their capacity to bind single-stranded (ss) and double-stranded (ds) DNA and RNA. Nucleic acid mobility shift occurred only with dsRNA in the presence of B2 and B2:GFP as indicated by white arrows **(A,C,E)** but not in the presence of B2m **(B,F)**. dsRNA used for EMSA was of bacteriophage Phi6 (Φ6) or synthetic origin except in **(F)** where low molecular weight dsRNA ladder was used for EMSA. In the latter case, clear mobility shift was restricted to dsRNA species longer than 30 bp **(F)**. Numbers below Φ6 lanes correspond to the amount (in ng) of B2, B2m and B2:GFP added in each sample. Acquisitions were performed under UV excitation for nucleic acid visualization **(A–C)** or at 488 nm for B2:GFP visualization **(D)**. **(E,F)** are composite images showing nucleic acids (white) and B2:GFP (Green). Ladders are indicated by L and corresponding sizes are given in kbp on the left sides except in **(F)** (bp).

Initially, we tested the capacity of FHV B2 residues 1-73 fused C-terminally to His_6_-StrepTagII (B2 hereafter) or to EGFP-His_6_-StrepTagII (B2:GFP, hereafter) to recognize specifically dsRNA *in vitro*. To do so, electromobility shift assays (EMSA) were performed in agarose gels (Figure 1). In agreement with the capacity of the full-length protein to specifically recognize dsRNA (Chao et al., 2005), nucleic acid mobility shifts were only observed with dsRNA in the presence of B2 or B2:GFP (Figure 1, white arrows), but not with dsDNA or single-stranded (ss) RNA or DNA (Figures 1A,**C–E**), nor when two key residues C44 and K47 of B2 involved in dsRNA recognition (Chao et al., 2005) were mutated (B2m, Figure 1B). We also show that dsRNA binding was sequence-independent, since electrophoretic mobility shifts occurred with both dsRNA of bacteriophage Phi6 (Φ6) or of synthetic origin (Figures 1A,C–E). Finally, using low molecular weight dsRNA ladder, we could show that B2 and B2:GFP preferentially bind dsRNA species longer than 30 bp since 21 and 30 bp dsRNA species were still visible upon addition of B2 and B2:GFP contrarily to higher molecular weight species (Figure 1F). No electromobility shift of dsRNA ladder was observed in the presence of mutated B2m (Figure 1F).

### Specific detection of dsRNA by northwestern blotting

**Figure 2:**
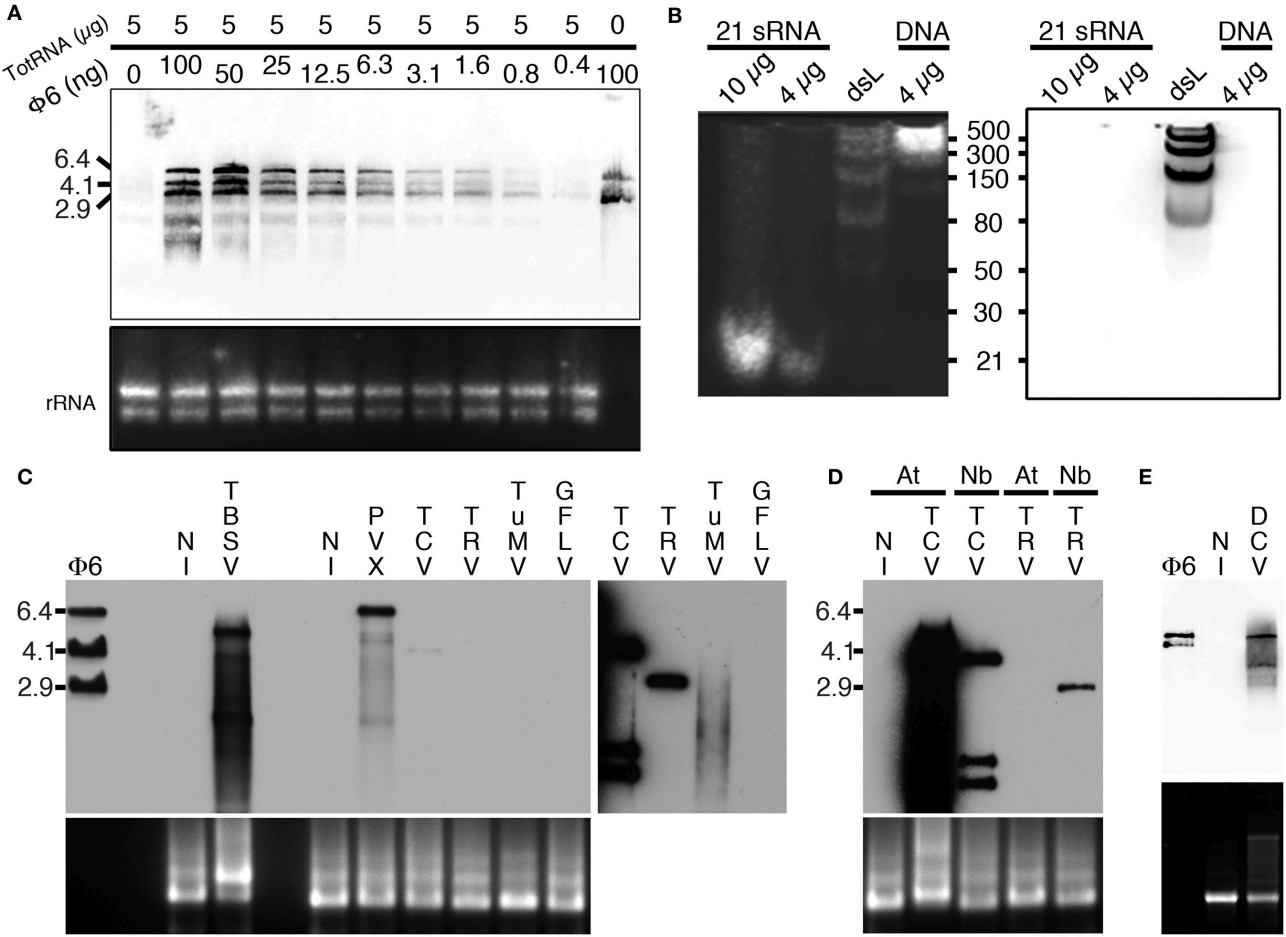
Specific detection of dsRNA by northwestern blotting. **(A)** B2 was tested by northwestern blotting (upper panel) for its capacity to specifically recognize dsRNA. For this decreasing amounts of dsRNA Φ6 (from 100 to 0.4 ng) in the presence of a constant concentration of total RNA from healthy *N. benthamiana* (5 μg per lane) were probed with B2. Note that up to 0.4 ng of dsRNA can be detected. **(B)** B2 was tested by northwestern blotting (right panel) for its capacity to specifically recognize small dsRNA species. For this, synthetic 21 bp dsRNA duplexes, dsRNA ladder or dsDNA ladder were probed with B2. Note that only dsRNA species with size superior to 50 bp could be detected. **(C)** B2 was tested by northwestern blotting (upper panel) for its capacity to specifically recognize viral dsRNA species. For this, 10 μg of total RNA extracted from systemically-infected TBSV- PVX- TCV- TRV- TuMV and GFLV-infected *N. benthamiana* leaves collected at 11–14 dpi were probed with B2. A short (1–2 s) and a long exposure (10 min) of the same membrane are presented side by side. Note dsRNA species of various sizes could be detected in all samples except in healthy (NI) and GFLV-infected ones. **(D)** Northwestern detection of dsRNA present in total RNA extracts from healthy or TCV and TRV systemically-infected *N. benthamiana* (Nb) and *A. thaliana* (At) leaves collected at 11–14 dpi. **(E)** Northwestern detection of dsRNA present in total RNA from healthy or DCV-infected S2 insect cells. Ethidium bromide-stained total RNA was used as loading control (lower panels except in **B**, left panel).

Considering the capacity of B2 to specifically recognize dsRNA *in vitro* by EMSA, we tested whether B2 could be used as a probe to specifically detect dsRNA by northwestern blotting (as detailed in the material and methods section). To do so, we initially tested the capacity of B2 to detect decreasing amounts of Φ6 dsRNA (from 100 to 0.4 ng) when mixed with 5 μg of total plant RNA (Figure 2A). Three major bands corresponding to the three genomic dsRNA species of bacteriophage Φ6 were detected with all dilutions except when dsRNA was absent (Figure 2A). Similarly, we tested the capacity of B2 to detect short dsRNA species by northwestern blotting (Figure 2B). In agreement with EMSA (Figure 1), 21bp dsRNA species remained undetectable *in vitro* (up to 10 μg were tested), similarly to 30 and 50 bp dsRNA species. Only dsRNA with size ≥80 bp were clearly detected on membranes. In view of our results, we then tested the capacity of B2 to detect dsRNA in total RNA extracts from systemically-infected *Nicotiana benthamiana* leaves. Virus used for inoculation included Tomato bushy stunt virus (TBSV) and Turnip crinkle virus (TCV) in the family *Tombusviridae*, Potato virus X (PVX) in the family *Alphaflexiviridae*, Tobacco rattle virus (TRV) in the family *Virgaviridae*, Turnip mosaic virus (TuMV) in the family *Potyviridae* and Grapevine fanleaf virus (GFLV) in the family *Secoviridae*. Upon short exposure (1–2 s), specific high molecular weight bands were clearly detected in TBSV- and PVX-infected samples and weakly with TCV (Figure 2C, left panel), but not in the sample from healthy *N. benthamiana* (NI, Figure 2C). Upon longer exposure (10 min), high molecular weight dsRNA bands could be seen in all infected samples except those infected with GFLV. In a similar manner, northwestern blotting revealed suitable for the detection of high molecular weight dsRNA species from TCV- but not TRV-systemically infected *Arabidopsis thaliana* leaf total RNA extracts (Figure 2D). Again, no signal was present in uninfected leaf extracts. We noticed that dsRNA species produced upon infection with TCV in systemically infected leaves were far more abundant in *Arabidopsis* than in *N. benthamiana*, likely reflecting differences in replication efficiency of TCV in the two hosts. Finally, northwestern blotting revealed also potent for the detection of dsRNA in total RNA extracts from drosophila C virus (DCV)-infected but not healthy S2 insect cells (Figure 2E). Altogether, our results demonstrate that northwestern blotting using B2 is a powerful technique to detect dsRNA species with size ≥80 bp and a limit of detection in the nanogram amount. In this manner, RNA-virus infected samples, whether of plant or animal origin, could be easily discriminated from healthy samples, making B2-northwestern blotting a robust method for broad-spectrum RNA-virus diagnostics. Negative detection of GFLV from *N. benthamiana* samples likely reflects the poor replication efficiency of this virus in this host contrarily to all other RNA viruses tested. Alternatively, weak or failed detection of dsRNA could reflect the transitory presence or partial production of this replication intermediate during infection as it has been shown for certain viruses such as potyvirus (Aramburu and Moreno, 1994; Lukács, 1994).

### Specific *in situ* detection of viral dsRNA by fluorescence labeling

**Figure 3:**
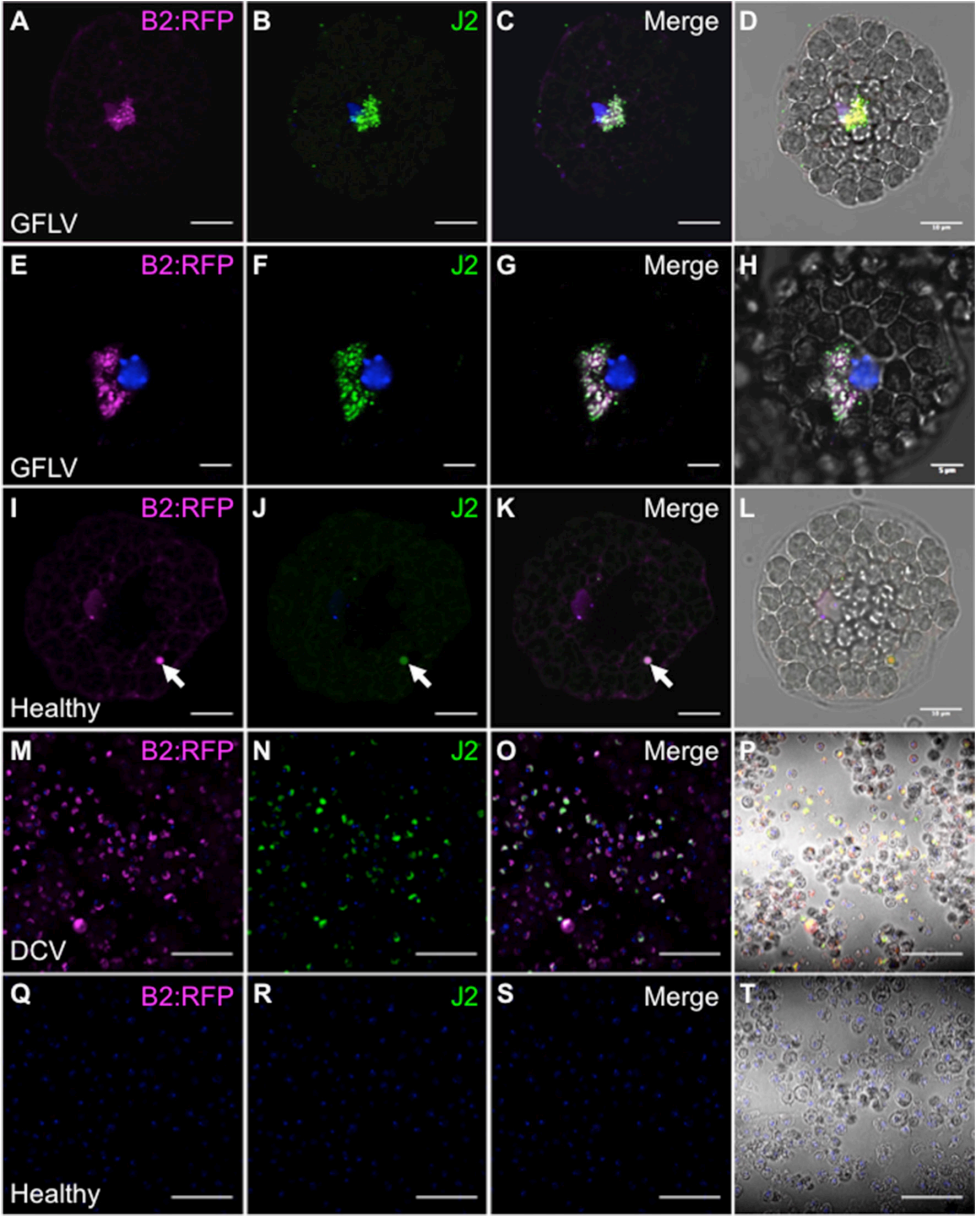
Specific detection of viral dsRNA-species by fluorescence labeling *in situ*. B2:RFP purified from *E. coli* and J2 mAb were used as fluorescent probes to detected viral dsRNA species in plant protoplasts **(A–L)** or in insect cells **(M–T)**. To this extent, GFLV infected **(A–H)** or healthy Arabidopsis protoplasts **(I–L)** as well as DCV-infected **(M–P)** or healthy insect cells **(Q–T)** were fluorescently labeled. Note that fluorescent labeling was restricted to infected samples where B2:RFP-labeling colocalized with J2-labeling. Arrowhead point to an autofluorescent structure seen upon fixation of protoplasts with glutaraldehyde. Scale bars: 5 μm **(E–H)**, 10 μm (**A–D, I–L**) and 50 μm **(M–T)**.

Similarly to northwestern blotting, we tested the capacity of a B2 genetically fused to TagRFP (B2:RFP) purified from *E. coli* to fluorescently label viral dsRNA species in fixed plant and insect cells. To do so, protoplasts isolated from healthy and GFLV-infected Arabidopsis as well as healthy and DCV-infected insect cells were co-incubated with B2:RFP and J2 mAb, a well-recognized dsRNA-specific marker (Schönborn et al., 1991). B2:RFP labeling was clearly observed in infected protoplast (Figures 3A–H) and insect (Figures 3M–P) samples contrarily to non-infected samples in which either very weak unspecific signal likely due to autofluorescence generated during glutaraldehyde fixation of protoplasts (Figures 3I–L, arrowheads) or no signal at all was detected (Figures 3Q–T). Importantly, in infected samples B2:RFP labeling colocalized nearly perfectly with J2 labeling, demonstrating that B2:RFP specifically labels dsRNA and is therefore an excellent substitute to J2 mAb. It is assumed that labeled dsRNA species correspond to cytoplasmic viral intermediates of replication present in viral factories (Ritzenthaler and Elamawi, 2006; Laliberté and Sanfaçon, 2010). Our results also indicate that fluorescence labeling is very sensitive since it allowed the detection of GFLV replication complexes in Arabidopsis protoplasts that looked very similar to those described in tobacco BY-2 cells (Ritzenthaler et al., 2002a) and that failed to be detected by northwestern blotting of systemically infected *N. benthamiana* samples (Figure 2C).

### Specific detection of viral dsRNA *in vivo*

**Figure 4:**
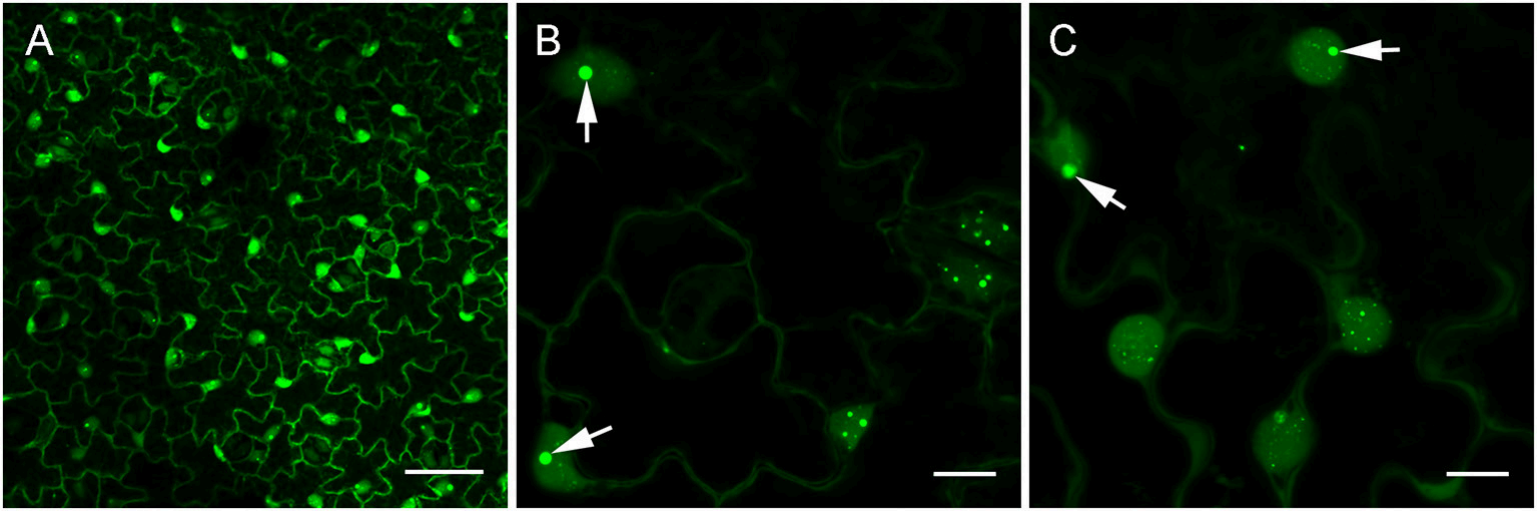
Confocal imaging of healthy dsRNA reporter *N. benthamiana*. Leaves from healthy dsRNA reporter *N. benthamiana* were observed at low **(A)** and high magnification **(B,C)**. Note the typical nucleo-cytoplasmic localization of B2:GFP in the leaf epidermal cells. At higher magnification, nuclear localization of B2:GFP appeared speckled and clearly enriched in the nucleoli (arrows in **B,C**). Scale bars: 50 μm **(A)** and 10 μm **(B,C)**.

**Figure 5:**
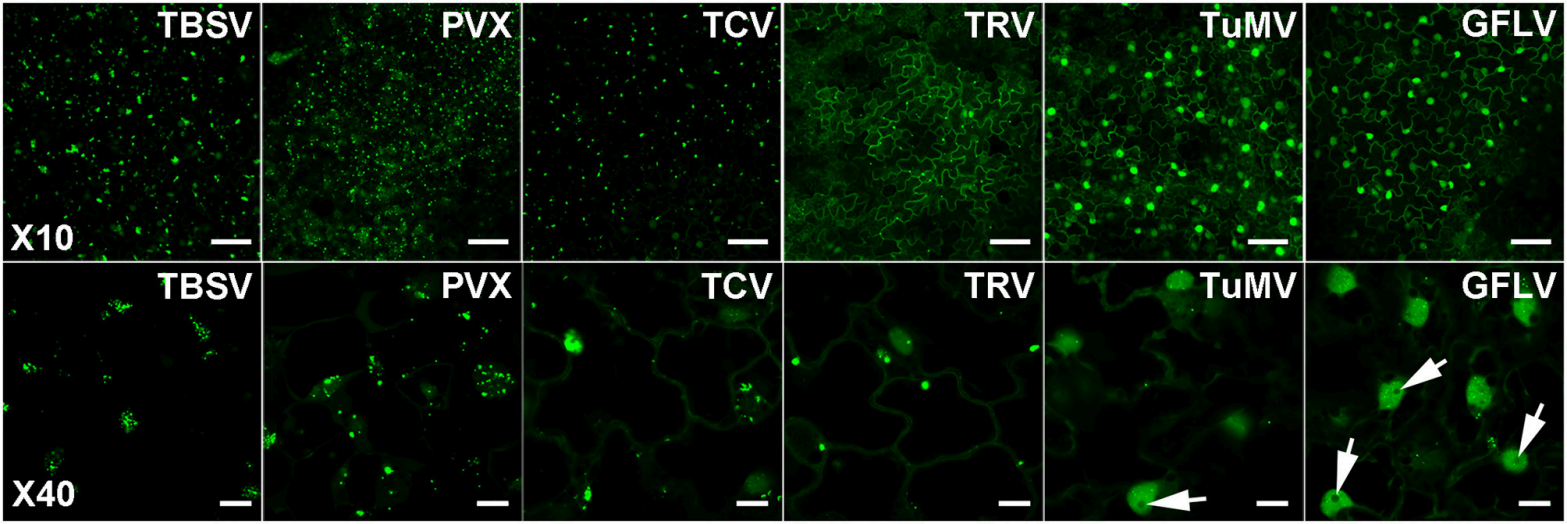
Confocal imaging of dsRNA reporter *N. benthamiana* infected with TBSV, PVX, TCV, TRV, TuMV, and GFLV. Virus infection resulted in a variety of patterns of intracellular relocation of B2:GFP. Profound modifications of B2:GFP localization to large cytoplasmic aggregates were observed upon TBSV and PVX infections. Smaller cytoplasmic aggregates were observed upon TCV and TRV-infections and almost no modification in B2:GFP localization occurred upon TuMV and GFLV infection except for the near depletion of B2:GFP from the nucleoli (arrows, compare with Figure 4). Scale bars: 50 μm (upper panels) and 10 μm (lower panels).

In view of our *in vitro* results and the report of dsRNA detection by dRBFC (Cheng et al., 2015), we generated transgenic *N. benthamiana* stably expressing B2 genetically fused to EGFP-His_6_-StrepTagII (B2:GFP) or TagRFP (B2:RFP) under the control of the 35S promoter. Confocal analysis of leaf epidermal cells at low magnification revealed a nucleo-cytoplasmic localization of B2:GFP (Figure 4A). At higher magnification, B2:GFP was found to be diffuse in the cytoplasm and highly enriched in the nucleolus and in nucleoplasmic speckles. Similar structures were observed by dRBFC (Cheng et al., 2015) and likely represent nuclear dicing bodies enriched in DCL1 and DRB4 (Figures 4B,C) (Fang and Spector, 2007; Nakazawa et al., 2007). Upon infection with TBSV, PVX, TCV, TRV, TuMV, or GFLV, confocal microscopy of inoculated leaves at 6-8 dpi revealed various patterns of B2:GFP localization (Figure 5). In TBSV or PVX infected leaves, B2:GFP localized to large cytoplasmic aggregates whereas smaller cytoplasmic aggregates or granules were observed with TCV and TRV. TBSV and PVX aggregates were clearly visible even at low magnification (Figure 5, upper panels). At higher magnification, the aggregates appeared polymorphic and frequently associated with a near complete depletion of B2:GFP from the nucleus (Figure 5, lower panels). In contrast, TuMV- and GFLV-infected samples were similar to uninfected plants (compare Figures 4, 5) with only small cytoplasmic granules observed with TuMV and none for GFLV, but remarkably, near-complete depletion of B2:GFP from nucleoli in both infections (Figure 5, arrows). These results are consistent with northwestern blots from infected *N. benthamiana* (Figure 2C), where detection of TuMV dsRNA was weak and B2 failed to detect any GFLV dsRNA.

**Figure 6:**
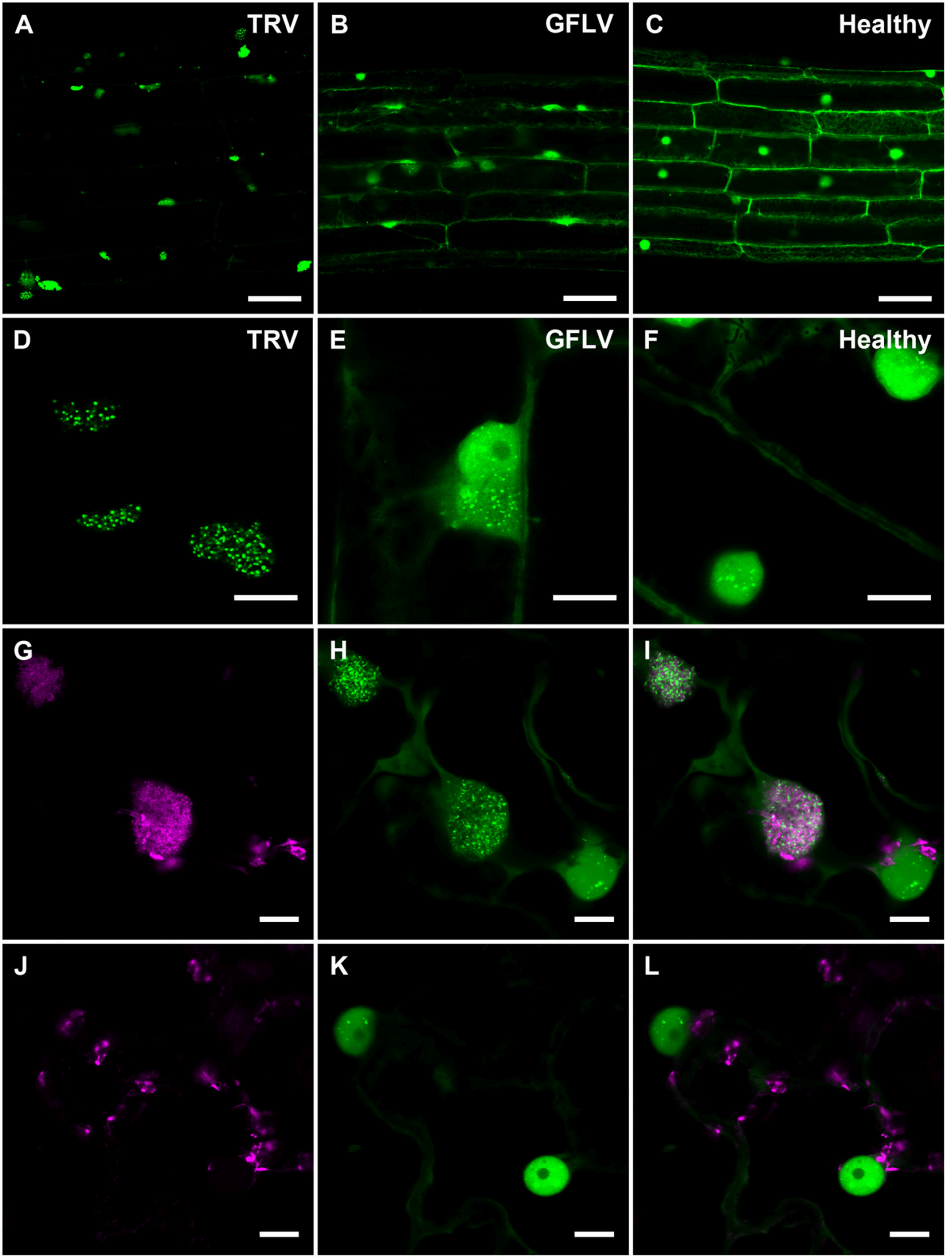
Confocal imaging of B2:GFP in roots from TRV- and GFLV-infected dsRNA reporter *N. benthamiana* and in TRV-infected leaves coexpressing B2:GFP and the mitochondrial marker F_0_-ATPase:RFP. **(A,D)** TRV-infected, **(B,E)** GFLV-infected and **(C,F)** healthy root cells constitutively expressing B2:GFP. Note the intensely and moderately labeled punctate replication complexes found in the cytoplasm of TRV-and GFLV-infected root cells, respectively. In healthy root cells no cytoplasmic aggregates can be observed. Intracellular localization of the mitochondrial marker F_0_-ATPase:RFP **(G,J)** and B2:GFP **(H,K)** in leaf epidermal cell of dsRNA reporter plants. **(I,L)** Corresponding merged images. **(G–I)** correspond to TRV-infected and **(J–L)** to healthy reporter plants. Scale bars: 50 μm **(A–C)** and 10 μm **(D–L)**.

Considering TRV and GFLV are transmitted by soil-borne nematodes (Schellenberger et al., 2011; MacFarlane et al., 2016; Andret-Link et al., 2017), we also investigated their effects on B2:GFP distribution in roots from transgenic *N. benthamiana* reporter plants (Figure 6). Similarly to leaf epidermal cells, TRV infection induced profound modifications in the intracellular B2:GFP localization with most of the fluorescence being depleted from nuclei and redistributed into large (10 μm or larger) cytoplasmic replication complexes in which B2:GFP was punctate (Figures 6A,D). These punctate cytoplasmic structures likely derive from, or correspond to mitochondria as deduced from the colocalization of B2:GFP with the mitochondrial marker F_0_-ATPase (Sieber et al., 2011; Michaud et al., 2014) in these structures (Figures 6G–I) that were never observed in cells from healthy plants (Figures 6J–L). This in agreement with previous reports indicating that TRV replicates in association with mitochondrial membranes (Otulak et al., 2015). Although the modifications induced by GFLV were milder than with TRV, B2:GFP-enriched cytoplasmic aggregates likely corresponding to replication complexes were also detected in roots cells from GFLV-infected reporter plants (Figures 6B–E). In contrast, cytoplasmic aggregates were absent from healthy root cells (Figures 6C,F). Altogether our results show that B2:GFP acts as a dsRNA marker not only *in vitro* but also *in vivo* upon stable expression in transgenic plants that act as reporter plants.

### Spatio-temporal analysis of PVX infection in *N. benthamiana* reporter plants

**Figure 7:**
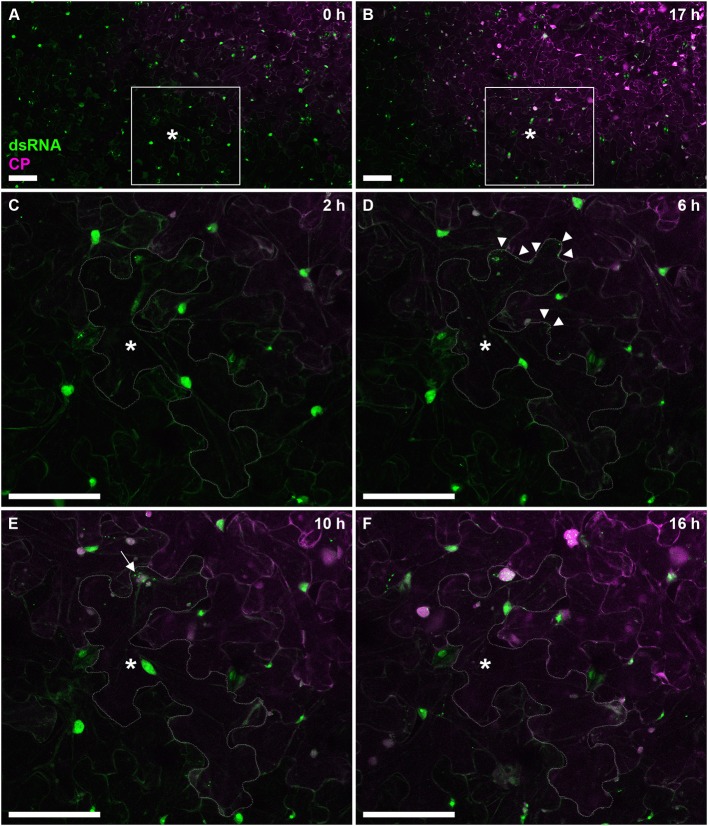
Time course of PVX infection. Progress of a PVX.mCherry-CP infection on a dsRNA reporter *N. benthamiana* leaf over 17 h. Images **(A,B)** give an overview of the infection front at 0 and 17 h post-infection, respectively. The boxed area is enlarged in images **(C–F)**, which were taken at the indicated time points. As the cell marked with an asterisk (^*^cell outline delineated in **C–F**) becomes infected, red fluorescence of mCherry-CP becomes detectable **(D)** and B2:GFP labels small peripheral replication sites at the side of the cell adjacent to an already infected cell (arrow tips). As the infection progresses, replication sites increase in size with one becoming dominant (**E**, arrow), and a neighboring cell shows the first faint signal from the mCherry-CP infection marker. About 10 h after the cell has become visibly infected **(F)**, the dominant replication site has developed into an X-body. Note that the nucleus has moved toward the X-body. All images are maximum projections of entire confocal z-stacks. Scale bars: 100 μm.

To further test the versatility of our dsRNA reporter transgenic *N. benthamiana* lines, we next followed progress of the recombinant PVX.mCherry-CP infection (Tilsner et al., 2009, 2012, 2013; Linnik et al., 2013) over a 17 h time period (Figure 7). The appearance of virus-encoded mCherry-CP coincided with detection of dsRNA in granular structures at the cell periphery adjacent to a neighboring infected cell (Figure 7D). Over the next 12 h, as the level of virus-produced mCherry-CP increased, dsRNA-containing structures became larger and one of them eventually developed into a dominant replication “factory,” the X-body (Tilsner et al., 2012; Figure 7F). About 4h after virus infection first became detectable, a neighboring cell in the direction of virus movement, showed the first faint traces of mCherry-CP (Figure 7E). This is faster than cell-to-cell movement of PVX as determined previously by trichome microinjections (8–10 h; Angell et al., 1996), however it has been reported for Tobacco mosaic virus that the speed of cell-to-cell movement increases from 18 to 20 h for the first cell-cell boundary to 3–4 for each subsequent one (Kawakami et al., 2004).

**Figure 8:**
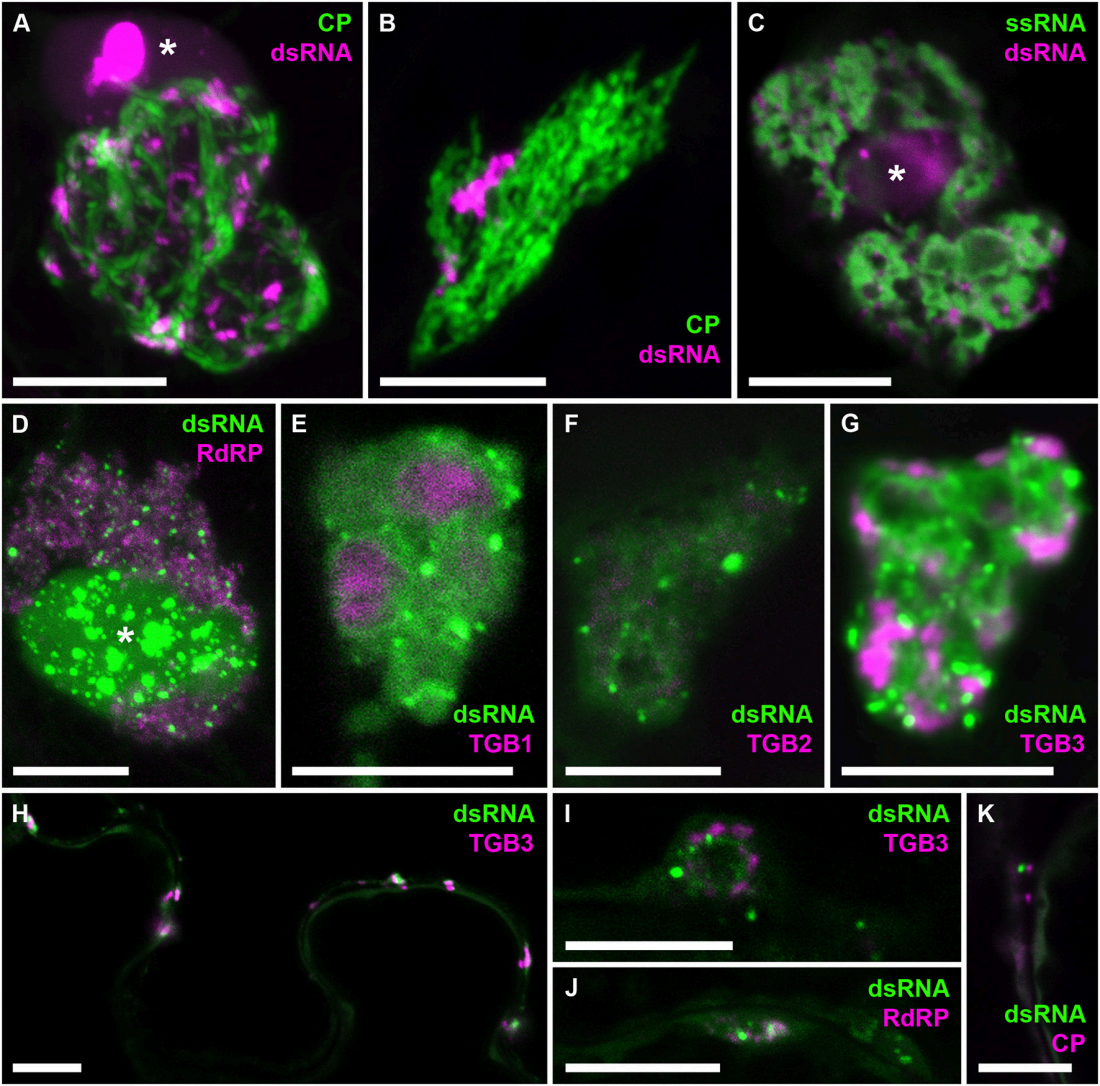
Co-localization of dsRNA with components of PVX infection. B2:RFP **(A–C)** or B2:GFP **(D–K)** dsRNA reporter in PVX-infected cells. **(A)** dsRNA granules within an X-body surrounded by GFP-CP-decorated virus particles. **(B)** The dsRNA reporter remains associated with virus particles in the absence of an X-body during infection with PVX.ΔTGB1.GFP-CP (Tilsner et al., 2012). **(C)** dsRNA-containing granules between “whorls” of single-stranded vRNA labeled by Pumilio-BiFC (Tilsner et al., 2009). **(D–G)** Within the X-body, none of the ectopically expressed viral proteins, truncated replicase (RdRP) or Triple Gene Block 1-3 (TGB1-3) co-localize with the dsRNA marker, although RdRP and TGB3 show granular locations within the X-body as well. Note that dsRNA is sometimes found in “whorls” similar to those observed for viral ssRNA **(E–G,I)**, which are surrounding aggregates of TGB1 protein **(E)** as described for ssRNA (Tilsner et al., 2009). **(H)** Association and partial co-localization of the dsRNA reporter with peripheral membrane structures labeled by TGB3, which have been shown to be associated with plasmodesmata (Tilsner et al., 2013). **(I,J)** Association of dsRNA marker with TGB3 and RdRP in peripheral replication sites. **(K)** Association of dsRNA marker with mCherry-CP labeled plasmodesmata. Where nuclei are visible in the images they are marked by an asterisk (^*^). Images **(A,B,D,J,K)** are maximum projections of entire confocal z-stacks whereas images **(C,E–I)** are individual confocal sections. Scale bars: 10 μm.

To analyze the distribution of dsRNA in PVX replication sites in more detail, we obtained high-resolution co-localizations with PVX-encoded proteins and also with viral ssRNA (Figure 8). As PVX replicase and movement proteins are either functionally impaired when fused to fluorescent proteins, or difficult to tag within the viral genome due to overlapping open reading frames, they were ectopically expressed in infected tissue in addition to the corresponding virus-expressed untagged proteins as in previous studies of the PVX replication sites (Tilsner et al., 2009, 2012, 2013; Linnik et al., 2013). dsRNA was associated with bundles of virus particles, which surround the X-body (Figure 8A). Interestingly, this association was maintained when the virus lacked a functional TGB1 protein (Figure 8B), and thus an organized X-body (Tilsner et al., 2012), indicating that virus particles may remain attached to replication sites in either case. Cheng et al. (2015) noticed that PVX dsRNA showed a more granular localization within the X-body compared to viral ssRNA observed with the Pumilio-BiFC system, which is found in typical circular “whorls” around aggregates of TGB1 protein (Tilsner et al., 2009). We obtained direct co-localization of both RNA reporters that generally supported this distinction. However, in some cases the dsRNA reporter also localized to “whorls” in addition to granules (Figures 8E–G,I). Currently it is not clear what these structures represent and why the localization of dsRNA can differ between different replication sites. None of the viral proteins showed a direct co-localization with dsRNA, despite RNA binding activities of the RdRP, TGB1, and TGB2 proteins, and co-localization of TGB3 with RdRP-containing replicative granules (Bamunusinghe et al., 2009). As the fluorescently labeled viral proteins were expressed ectopically, we cannot exclude that their unlabeled counterparts, expressed in *cis* from the viral genome, did co-localize with dsRNA. It is also possible that viral replication intermediates are not easily accessible to most ectopically expressed proteins, with RdRP-RFP and TGB1-RFP much larger than the B2:FP dsRNA reporter and TGB2 and TGB3 fusions membrane-bound. However, dsRNA was associated with all of the viral proteins, both in the large X-bodies and in smaller peripheral replication sites (Figures 8H–K), which may be associated with plasmodesmata (Figure 8K) and participate in co-replicational virus transport (Tilsner et al., 2013).

### Detection of GFLV and TuMV replication complexes

**Figure 9:**
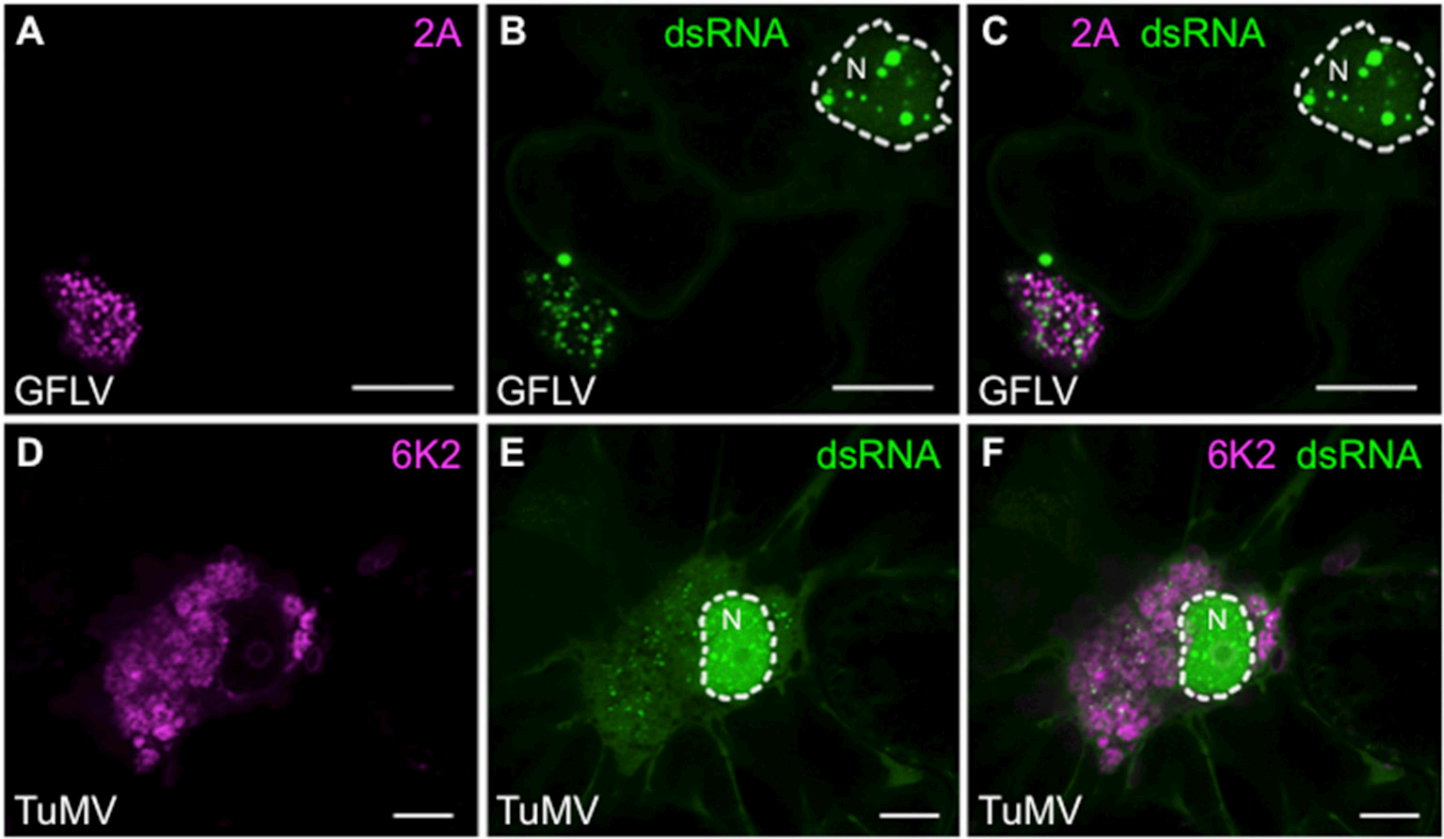
Colocalization of dsRNA with 2A protein from GFLV and 6K2 protein from TuMV. **(A)** 2A:TagRFP and **(B)** B2:GFP labeled replication complexes in the cytoplasm of GFLV-TagRFP-infected leaf epidermal cell of dsRNA reporter plant. **(C)** Merged (**A**+**B**) images. **(D)** 6K2:mCherry and **(E)** B2:GFP labeled replication complexes in the cytoplasm of TuMV.6K2:mCherry-infected leaf epidermal cell of dsRNA reporter plant. The nuclei (N) are delineated. **(F)** Merged (**D**+**E**) images. Scale bars: 5 μm.

Finally, considering that dsRNA reporter plants did not reveal significant modifications of B2:GFP intracellular distribution upon infection with TuMV and GFLV, apart from the depletion of the reporter from nucleoli (Figure 5), we analyzed these infections in more detail using the recombinant versions of GFLV-TagRFP in which 2A protein is fused to TagRFP (Hemmer et al., 2017) and TuMV.6K2:mCherry (Grangeon et al., 2013). Both 2A from GFLV and 6K2 from TuMV are well-described markers of the cytoplasmic viral replication factories produced by the two viruses. Although more modest than with TBSV and PVX, clear redistribution of B2:GFP to replication factories was also detected upon infection with GFLV (Figures 9A–C) and with TuMV (Figures 9D–F). In agreement with Gaire et al. (1999) and (Ritzenthaler et al., 2002a), GFLV replication factories appeared as well-defined punctate 2A-labeled clusters in which dsRNA was clearly detected. In the case of TuMV, perinuclear 6K2-labeled replication factories formed small whorls in which discreet dsRNA labeling was detected. With both GFLV and TuMV, B2:GFP was still clearly present within the nucleus, either as well-defined speckles (GFLV, Figures 9B,C) or numerous punctate structures (TuMV, Figures 9E,F), but depleted from the nucleolus.

## Discussion

In the repertoire of techniques that have been employed to detect long dsRNA, immunological approaches have proven to be particularly powerful for the identification of viral dsRNA *in vitro* (Poynter and DeWitte-Orr, 2017). Thus, the use of dsRNA-specific antibodies, in particular the J2 mAb (Schönborn et al., 1991), in ELISA, immunoblotting, dot blot, immunofluorescence, immunohistochemistry and immunoprecipitation has allowed the detection or visualization of a whole range of viral dsRNA complexes produced by RNA- and DNA-viruses infecting eukaryotes or prokaryotes and covered in hundreds of publications. This is in sharp contrast with *in vivo* studies that remain scarce and limited essentially to plants where the recently developed BiFC approach dRBFC uses well-characterized dsRNA binding domains from viral DRBs as dsRNA-specific molecular probes (Cheng et al., 2015; Li et al., 2016; Xu and Nagy, 2016; Zhang et al., 2017).

Here, we show that the 73 amino acid dsRNA binding domain from the FHV B2 protein (Chao et al., 2005) acts as a dsRNA-specific molecular probe both *in vitro* and *in vivo*. *In vitro*, the northwestern blotting and fluorescence labeling methods which we developed proved to be highly efficient for dsRNA detection in complex samples such as total RNA or fixed cells from infected plants or insects. The detection threshold of long dsRNA in northwestern blotting, which is approximately 1 ng, proved to be compatible with the rapid and efficient detection of a range of plant and insect RNA viruses including TBSV, TCV, TRV, TuMV, and DCV. Only GFLV, a virus that replicates with low efficiency (Pinck et al., 1988) failed to be detected in our northwestern assays. However, this limitation could be circumvented by *in situ* fluorescence labeling using B2 fused to fluorescent proteins as dsRNA probe similarly to immunofluorescence labeling using dsRNA specific antibodies. In this case, GFLV and DCV replication factories could be easily identified and colocalized with the J2-labeled dsRNA. Altogether, we show here that B2 is an excellent substitute to dsRNA mAb such as J2 both for northwerstern and fluorescence labeling. Furthermore, this system is not hampered by the classical limitations associated to mAb use such as chemical coupling to fluorophores, production yield and of course cost. In fact, in our hands, B2, whether fused to His_6−_StrepTagII or to fluorescent proteins, was easily produced and purified from *E. coli* in milligram amounts.

*In vivo*, fluorescent fusions of B2 expressed in stable transgenic reporter plants enabled detection of infection by all tested viruses through changes in fluorescence localization. This was true even for GFLV, which was undetectable by northwestern blotting but still produced a depletion of B2:GFP from the nucleolus. This could be due to indirect effects of the virus on nuclear metabolism such as silencing or host gene shut off (Wang and Maule, 1995; Aranda and Maule, 1998) rather than direct recruitment of the reporter by viral dsRNA. Irrespective of the underlying mechanism, the dsRNA reporter plants will be useful for detection of virus infections, for instance to validate decontamination procedures of infected plant material. Additionally, the reporter plants also allowed the detailed study of viral infection processes *in vivo*. We demonstrate here that the dsRNA reporter plants are suitable for time course experiments, which can be carried out over extended periods because of the stable transgenic expression of B2:FP, and subcellular co-localizations. This provides a substantial advantage over dRBFC that involves transient expression of two components and is therefore limited to plant tissues that can be agroinfiltrated such as *N. benthamiana* leaves. Although comparison was not made here, it is clear that the signal-to-noise ratio generated by dRBFC (Cheng et al., 2015) vs. constitutive B2-GFP expression is *a priori* very much in favor of dRBFC. Nevertheless, we demonstrate that the signal-to-noise ratio observed in transgenic *N. benthamiana* is fully compatible with the visualization of dsRNA structures *in vivo*, particularly those produced during replication of positive-stranded RNA viruses. This includes viruses that produce large amounts of dsRNA (i.e., TBSV, PVX, see Figure 2) but also viruses that replicate far less efficiently and produce low amounts of dsRNA (i.e., TuMV, Figure 2) or even below detection levels dsRNA as determined by northwestern blotting (i.e., GFLV, Figure 2). In addition, we show that virus replication complexes visualization is not limited to leaves as with dRBFC but virtually any organ as illustrated with TRV- and GFLV-infected root tissue which may be exploited to gain novel insights into the transmission of this virus by soil-borne nematodes.

The different distributions of dsRNA observed for different viruses are likely reflective of the different composition and compartmentalization of their replication sites and can form the basis for further investigations using viral and host protein markers, cellular/metabolic inhibitors, etc. As examples we have colocalized TRV dsRNA with a mitochondrial marker, as well as PVX dsRNA with all five proteins encoded by this virus, as well as viral ssRNA. These latter data show a clear association of dsRNA with perinuclear and peripheral replication sites and indicate a possible temporal dynamic of how the replicative intermediates are distributed in viral “factories.” These studies could be complemented by co-localizations with markers of host organelles (Tilsner and Oparka, 2012). Overall, we expect that the dsRNA reporter plants will be of great utility to plant virus researchers, and we are planning to establish similar stable lines also in relevant animal/mammalian cell cultures.

In summary, we have shown that the B2 dsRNA-binding domain can be used to detect long dsRNA *in vitro* equally well as the commonly employed J2 antibody. Unlike J2, however, B2 is also suitable for detailed *in vivo* studies.

## Authors contributions

Conceived and designed the experiments: BM, MI, KH, VP, AG, PD, LD, JT, and CR. Performed the experiments: BM, MI, KH, VP, AG, LD, and JT. Analyzed the data: BM, MI, KH, VP, AG, PD, LD, JT, and CR. Wrote the paper: BM, MI, PD, LD, JT, and CR. First draft of the manuscript was edited and approved by all authors.

### Conflict of interest statement

The authors declare that the research was conducted in the absence of any commercial or financial relationships that could be construed as a potential conflict of interest.
