# Supplementary material for: Efficient Detection of Long dsRNA in Vitro and in Vivo Using the dsRNA Binding Domain from FHV B2 Protein
Source: Front Plant Sci. 2018 Feb 1;9:70. doi: 10.3389/fpls.2018.00070 (PMC5799278; doi:10.3389/fpls.2018.00070)
Supplement: Supplementary file 1 [file Table1.pdf]

Table S1: Primer sequences from 5’ to 3’ ends

#1	tgaaatcatatgccaagcaaactcgcgctaa
#2	tttattctcgagttatttttcaaattgaggatgagaccagtgatggtgatggtggtgggcctttccctctaggtatgcc
#3	aaacgcggcaaaactaacggtaagtc
#4	aggctagcgtgcaggttgt
#5	ggggacaagtttgtacaaaaaagcaggcttcatgccaagcaaactcgcgctaa
#6	caccatacctccaccagatccacctccggcctttccctctaggtatgcc
#7	aaggccggaggtggatctggtggaggtatggtgagcaagggcgag
#8	tactctgaattctattagtgatgatgatggtggtgcttgtacagctcgtccat
#9	gctcatacctccaccagatccacctccggcctttccctctaggtatgcc
#10	aaggccggaggtggatctggtggaggtatgagcgagctgattaaggaga
#11	gtgatggtgatggtggtgattgtgccccagtttgctag
#12	ggggaccactttgtacaagaaagctgggtcttatttttcaaattgaggatgagaccagtgatggtgatggtggtg
#13	ggggacaagtttgtacaaaaaagcaggcttcgaaggagatagaaccatgccaagcaaactcgcgctaa
#14	ggggaccactttgtacaagaaagctgggtcttagtgatggtgatggtggtg
